# Righteousness (and lefteousness) of the old brain

**DOI:** 10.18632/aging.102127

**Published:** 2019-07-23

**Authors:** Madalena Esteves, Nuno Sousa, Hugo Leite-Almeida

**Affiliations:** 1 Life and Health Sciences Research Institute (ICVS), School of Medicine, University of Minho, Campus de Gualtar, Braga 4710-057, Portugal; 2 ICVS/3B’s, PT Government Associate Laboratory, Braga/Guimarães, Portugal; 3 Clinical Academic Center, Braga, Portugal

**Keywords:** laterality, asymmetrical plasticity, aging, cognition, neuroimaging

Asymmetries are widespread across the animal kingdom, manifesting in a number of behaviors among which handedness/paw preference is the most recognizable – see for instance [1]. Such reflects an asymmetric organization of the CNS that can be found in virtually all levels of analysis including at the macro level the Yakovelovian torque (an anticlockwise torsion of the brain axis), the petalia (protrusions of the right frontal and left occipital cortices) and lateral ventricle asymmetry (L>R) as well as at the micro level regional volumetric (planum temporale’s minicolumns L>R) and cytometric asymmetries. At a functional level, asymmetric activations have been repeatedly reported in imaging studies. To what degree such reflects hemispheric specialization or simply an asymmetrical load it is not entirely clear. The pioneer observations of Paul Broca and Marc Dax in the mid 1800’s on aphasic patients with unilateral strokes point toward the first view. However, in aged individuals, bilateral activation is often associated with better performance in cognitive tasks, suggesting that in specific circumstances the two hemispheres concur for the same function. Such observation is at the core of the Hemispheric Asymmetry Reduction in Older Adults (HAROLD) model posing that contralateral networks homotopic to the normally activated region compensate for age-related loss of function [2]. Indeed, our lab has previously observed that, during a working memory task, recruitment of brain regions was mostly symmetrical in an aged population [3]. However, we also observed that left>right superior parietal activation was associated with better performance [3], which raises questions regarding the range of validity of this idea, and/or of the additional factors to be accounted for.

The aged brain is indeed of particular interest in the context of laterality. In a recent study, we observed that for most of the cortical and subcortical areas analyzed, a leftward or rightward bias was present [4]. Except for the fusiform gyrus in which a sex-dependent association was observed (increased leftward laterality index in females), all other were independent of sex, education, cognitive performance (high vs low performers) and age (51 to 82 years old). Interestingly, much less morphological asymmetries were found in another study using younger subjects ([5]; age range 19-40 years-old), though the pattern region/directionality was similar [5], suggesting an increase of morphological lateralization with age. In our study, asymmetries were associated with better learning and memory, inhibition/cognitive flexibility, verbal fluency and mood, though often mediated by sex and education. Eighteen months later, part of this population was reevaluated and all areas maintained their lateralization magnitude and direction [6]. However, in subcortical regions the manifestation of extreme variants, i.e. individuals whose LI varied most between the two evaluations, was significantly higher. The reason for this higher prevalence of extreme variation in subcortical regions is not clear. It is known that LI variation occurred as a result of volume alterations in both hemispheres (in opposing directions), suggesting that the phenomena was coordinated and physiologically relevant; also, it excluded potential local alterations of pathological (or other) nature. In fact, comparison of psychometric data from the two moments demonstrated that an improvement in Stroop interference score was associated with a thalamus leftward and caudate rightward volume variation. Additionally, a decrease in the Mini-Mental State Examination (MMSE; general cognition) was associated with increased rightward caudate volume.

While the volume-activation-behavior link is not fully established, when assessing the most lateralized function – language -, this flow seems exceedingly clear: the majority of the population has left>right language-related (peri-Sylvian) regions [7], which are activated in a left>right fashion during language-related tasks [7], which in turn associates with better performance [8]. From the above-mentioned volumetric studies one can infer that, in general, both structural and functional laterality may increase with age, and that exceptions (symmetry) are associated with contralateral compensation. Indeed, we might think of these structural asymmetries as a sort of an imprint of its past activity which, as we have seen, tends to be asymmetrical. As a general rule we found that asymmetry is associated with better cognitive performance and mood scores and in specific cases, the directionality of the asymmetry was even irrelevant – e.g. working memory/executive function performance (Digits Span Test backward) and total gray matter [4]. Also, some of the associations between psychometric and volumetric data were mediated by the number of years of education, which suggests a potential role of asymmetry in a cognitive reserve-like maintenance of function throughout aging. The causality between asymmetry and improved function remains however, elusive. Hypotheses raised so far suggest reduced processing time due to specialization, decreased competition and interference between homotopic regions but, again, this is in the realm of speculation. What we can agree upon is that lateralization is plastic. Even in a relatively short time windows it entails alterations in cognitive function and mood; if as a cause or a consequence of, it is not yet known.
